# Targeting the complex I and III of mitochondrial electron transport chain as a potentially viable option in liver cancer management

**DOI:** 10.1038/s41420-021-00675-x

**Published:** 2021-10-14

**Authors:** Qin Yang, Ling Wang, Jiaye Liu, Wanlu Cao, Qiuwei Pan, Meng Li

**Affiliations:** 1 Department of general surgery, The Third People’s Hospital of Chengdu & the Affiliated Hospital of Southwest Jiaotong University & the second medical school of Chengdu affiliated to Chongqing medical university, Chengdu, Sichuan 610031 China; 2 Erasmus MC Cancer Institute, Erasmus MC-University Medical Center Rotterdam, Rotterdam, The Netherlands; 3 Guangzhou Institutes of Biomedicine and Health, Chinese Academy of Sciences, Guangzhou, 510530 China; 4 Guangzhou Regenerative Medicine and Health Guangdong Laboratory, Bioland Laboratory, Guangzhou, 510005 China

**Keywords:** Cancer metabolism, Targeted therapies

## Abstract

Liver cancer is one of the most common and lethal types of oncological disease in the world, with limited treatment options. New treatment modalities are desperately needed, but their development is hampered by a lack of insight into the underlying molecular mechanisms of disease. It is clear that metabolic reprogramming in mitochondrial function is intimately linked to the liver cancer process, prompting the possibility to explore mitochondrial biochemistry as a potential therapeutic target. Here we report that depletion of mitochondrial DNA, pharmacologic inhibition of mitochondrial electron transport chain (mETC) complex I/complex III, or genetic of mETC complex I restricts cancer cell growth and clonogenicity in various preclinical models of liver cancer, including cell lines, mouse liver organoids, and murine xenografts. The restriction is linked to the production of reactive oxygen species, apoptosis induction and reduced ATP generation. As a result, our findings suggest that the mETC compartment of mitochondria could be a potential therapeutic target in liver cancer.

## Introduction

Liver cancer, mainly consisting of hepatocellular carcinoma (HCC) and cholangiocarcinoma (CCA), is the sixth most common cancer and one of the leading causes of cancer-related death [1]. Hepatitis B and C, as well as metabolic syndrome and type II diabetes, are all risk factors for HCC. The molecular details of disease progression and how these risk factors translate in cancer development are less clear, hampering rational efforts to develop new therapy. Apart from surgery, there are no curative options available, and clinical management of HCC is hampered by high recurrence rates and the resistance to systemic chemotherapy [2]. As a result, a greater understanding of the progression of liver cancer is urgently required.

Hepatocytes are a unique cell type in many aspects, including the high abundance of mitochondria in their cytoplasm, which appear to be involved in the oncogenic transformation of this cell type [3]. Liver cancer progression is accompanied by a series of well-defined genetic alterations concomitantly with cellular metabolic reprogramming [1, 4]. The central role of mitochondria in hepatocyte metabolism has given rise to speculation that this organelle may provide novel therapeutic targets for liver cancer treatment, despite the fact that our understanding of its functionality in the HCC process is far from complete [5].

Mitochondria are multi-membrane structures with their own DNA and translational machinery, though the mitochondrial function is critically dependent on import of proteins from the cytosol [6, 7]. The most central element in mitochondrial biochemistry is the mitochondrial electron transport chain (mETC), which is located in the inner mitochondrial membrane and consists of four complexes involved in oxidative phosphorylation [8]. Encouragingly, chronic use of metformin-like drugs that target mETC complex I reduces the risk for HCC development, which may also relate to the role of mETC in energy production, membrane potential maintenance, and redox balance control [9–12]. Studies indicated multiple mechanisms of action of metformin is involved in HCC inhibition, including activating adenosine monophosphate kinase (AMPK) to regulate metabolism and targeting mitochondrial complex I alterations in cellular energy metabolism and oxidative phosphorylation [13–15].

However, more direct studies on the importance of mETC in HCC are required to better determine the promise of mETC-directed therapy in liver cancer.

We attempted to investigate the significance of mETC components in liver cancer in response to the aforementioned considerations. The role of mETC complex I and III, as well as mitochondrial DNA in HCC physiology, was identified. The possibility of therapeutically targeting the mETC in liver cancer cells was investigated, which may guide the development of novel rational avenues for the treatment of HCC.

## Results

### Growth of liver cancer cell lines requires functional mETC complex I and III

**Fig. 1:**
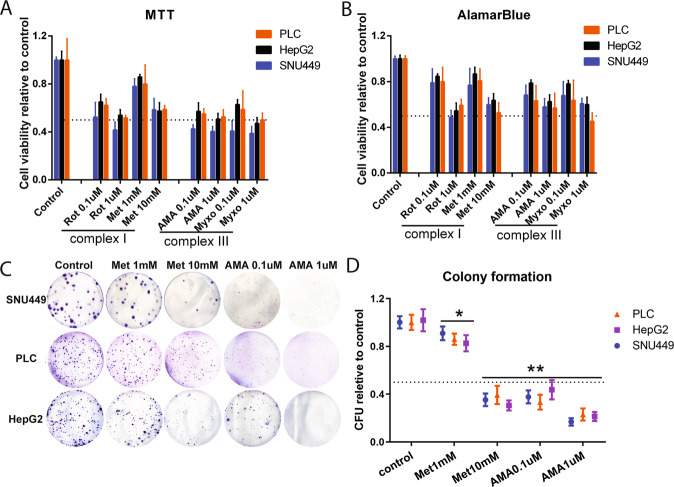
Complex I and III inhibitors of mitochondrial electron transport chain (mETC) inhibited growth of HCC cells. **A**, **B** HepG2, PLC/PRF/5, and SNU-449 cells were treated for 48 h with the mETC complex I inhibitors (ROT Rotenone, Met Metformin) and complex III inhibitors (AMA Antimycin A, MYXO Myxothiazol) at different concentrations. Cell viability was quantified by MTT and Alamar Blue (*N* = 9). **C**, **D** HepG2, PLC/PRF/5, and SNU-449 cells were treated with Met and AMA at different concentrations, and the colony formation efficiency was calculated (*N* = 9). *P*-value was calculated by comparing with corresponding controls. Results are expressed as mean ± SD, and group differences were tested by Mann–Whitney *U* test.

To study the effects of targeting mETC complexes in liver cancer, SNU-449, PLC/PRF/5 and HepG2 liver cancer cell lines were challenged with six pharmacological inhibitors targeting the four mETC complexes (I, II, III, and IV). Pharmacological inhibition of either mETC complex I (using ROT or Metformin) or III (using AMA or MYXO) reduced cell viability in all three liver cancer cell lines at two concentrations (Fig. 1A, B). While neither complex II (using TTFA) nor complex IV (using KCN) inhibitors showed obvious effects in this respect (supplementary Fig. S1).

We then assessed colony formation, pharmacological inhibition of either mETC complex I or mETC complex III reduced the colony formation efficiency of all three cell lines to <50% as compared to untreated controls (Fig. 1C, D), which was consistent with the results of cell survival. These results suggested that the functionality of mETC complex I and mETC complex III was required for liver cancer cell survival.

### Targeting mETC complex I and III inhibited the growth of mouse liver tumor organoids

**Fig. 2:**
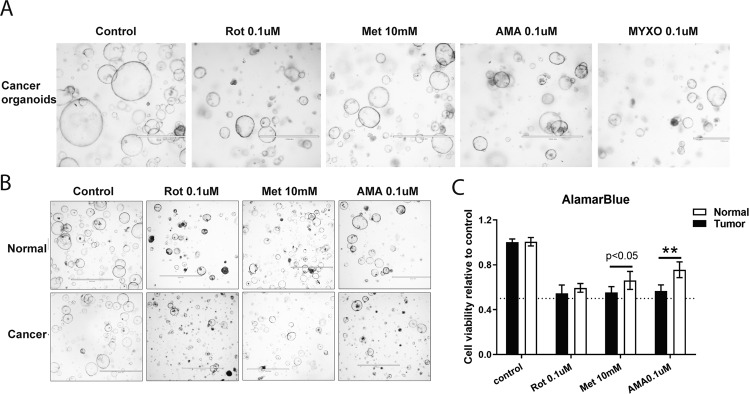
mETC complex I and III inhibitors impaired the growth of mouse liver tumor organoids. **A** Optical microscope images of liver tumor organoids after treating with the mETC complex I inhibitors (Rotenone, ROT; Metformin, Met) and complex III inhibitors (Antimycin A, AMA; Myxothiazol, MYXO) for 48 h (*N* = 3). **B** Optical microscope images of liver tumor organoids and normal liver organoids after treating with AMA, Met, and ROT for 48 h (*N* = 9). **C** The growth rates of tumor and normal liver organoids were measured by Alamar Blue assays (*N* = 9). Results are presented as mean ± SD, and group differences were tested by Mann–Whitney *U* test.

Although cell line models allow for rapid screening of cancer drugs, they only partly capture the cancer process. Experimentation with cancer organoids is cumbersome but more closely recapitulates the pathophysiological features of natural tumorigenesis, and it was recently shown that such organoids are a useful approach for studying liver cancer [16]. Hence, we further studied the effects of pharmacological inhibitors targeting mETC complex I and III in tumor organoids isolated from DEN-induced primary murine liver cancers compared to organoids derived from the healthy liver [17]. All complex I and III inhibitors (ROT, Met, AMA, and MYXO) limited the growth of both tumor organoids and untransformed organoids, but cancer organoids were more sensitive to such inhibition (Fig. 2A, B). In summary, the inhibition effects of pharmacological complex I and III inhibitors observed in cell lines were also recaptured in liver cancer organoids. These results illustrated that liver cancer was more sensitive to mETC inhibition than untransformed liver-derived material.

### Depletion of mitochondrial DNA or knockdown of mETC constituting proteins counteracts liver cancer cell survival

**Fig. 3:**
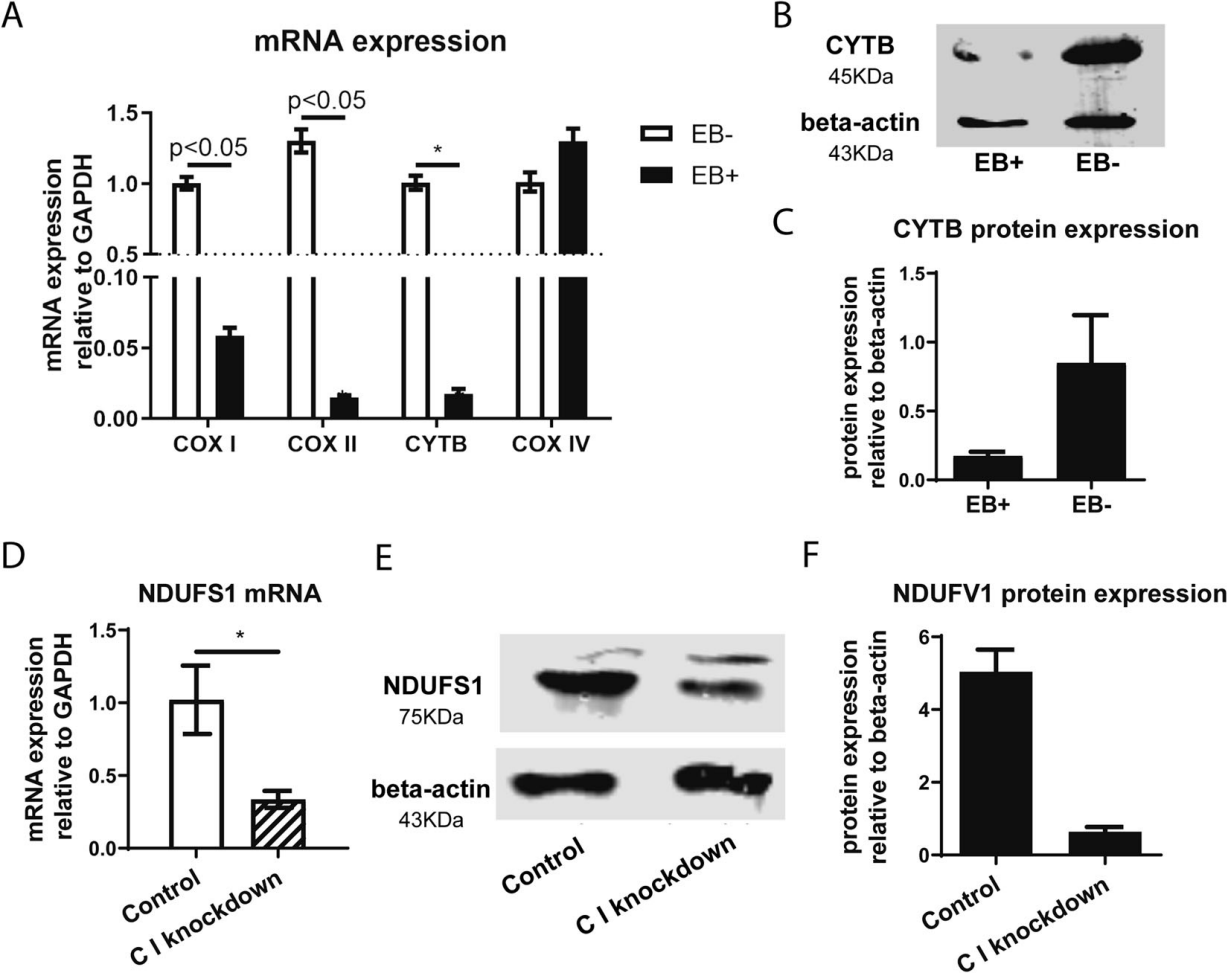
Establishing the mETC-deficient cell models. **A**–**C** Real-time PCR analysis of COX I, COX II, COX IV and CYTB mRNA (**A**) (*N* = 6) and Western blotting analysis of CYTB protein (**B**, **C**) (*N* = 3) in HCC cells after treatment with EB for 5 days. **D**–**F** Real-time PCR analysis of NDUFS1 mRNA (**D**) (*N* = 6) and Western blotting analysis of NDUFS1 protein (**E**, **F**) (*N* = 3) in HCC cells after transfected with NDUFS1 lentivirus. Results are presented as mean ± SD, group differences were tested by Mann–Whitney *U* test.

The importance of mETC integrity for liver cancer cell survival was then confirmed by two experimentally independent approaches. In the first approach, we depleted mitochondrial DNA from cells. Depletion of mitochondrial DNA impacts encoding of proteins that are part of the mETC, namely the cytochrome c oxidase subunit I (COX I), cytochrome c oxidase subunit II (COX II), and cytochrome oxidase (CYTB). Depletion of mitochondrial DNA by exposing PLC/PRF/5 cells to ethidium bromide (EB) strongly reduced expression of COX I, COX II, and CYTB, whereas expression of cytochrome c oxidase subunit IV (COX IV; which is encoded by nuclear DNA) was not affected (Fig. 3A–C). In the second approach, lentiviral vector-delivered shRNA was used to knockdown the mETC complex I subunit NDUFS1. The technical success of this strategy was documented in Fig. 3D–F. Both approaches were used to determine the effect of mETC integrity on liver cancer cell physiology.

**Fig. 4:**
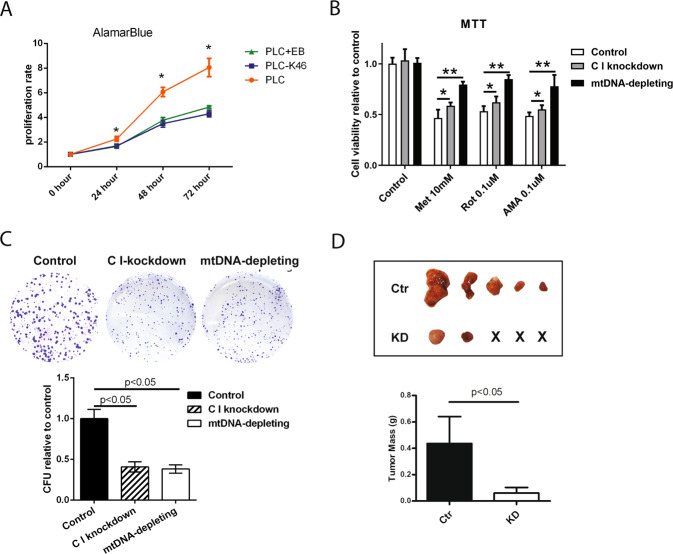
Compromising mETC integrity inhibits HCC cell growth in vitro. **A** Alamar Blue assays measure the cell growth rates in different liver cell lines (PLC/PRF/5, C I knockdown, and mtDNA depleting) were performed (*N* = 8). **B** MTT assays to test the mETC complex I and III inhibitors (Met, ROT, and AMA) effect on the proliferation of the three cell lines (*N* = 9). **C** Representative images of colony formation assay of PLC/PRF/5, C I knockdown, and mtDNA-depleting HCC cells, and the colony formation units (CFU) were calculated. The results were normalized to the control (PLC/PRF/5), setting to 100% plating efficiency (*N* = 4). **D** Ctr (PLC/PRF/5 control group) and KD (PLC/PRF/5 C I knockdown group) cells were injected subcutaneously into nude mice. Tumors were harvested from nude mice and weighed (*N* = 5). Results were presented as mean ± SD, and group differences were tested by Mann–Whitney *U* test or one-way ANOVA when appropriate; *p < 0.05.

Furthermore, both mETC targeting strategies diminished proliferation (Fig. 4A, B) and colony formation efficiency (Fig. 4C). However, compared with mitochondrial DNA depletion or NDUFS1 knockdown, pharmacological inhibitors of complex I were less effective in reducing cell viability (Fig. 4B). Upon subcutaneous engraftment in immunodeficient nude mice, cells with NDUFS1 knockdown were less capable of forming tumor in vivo compared with correspondence control (Fig. 4D). These results suggested that the reduction in liver cancer cell viability introduced by mETC inhibitors was on the basis of the integrity of the mETC.

### Targeting mETC complex I and III induced cell apoptosis

**Fig. 5:**
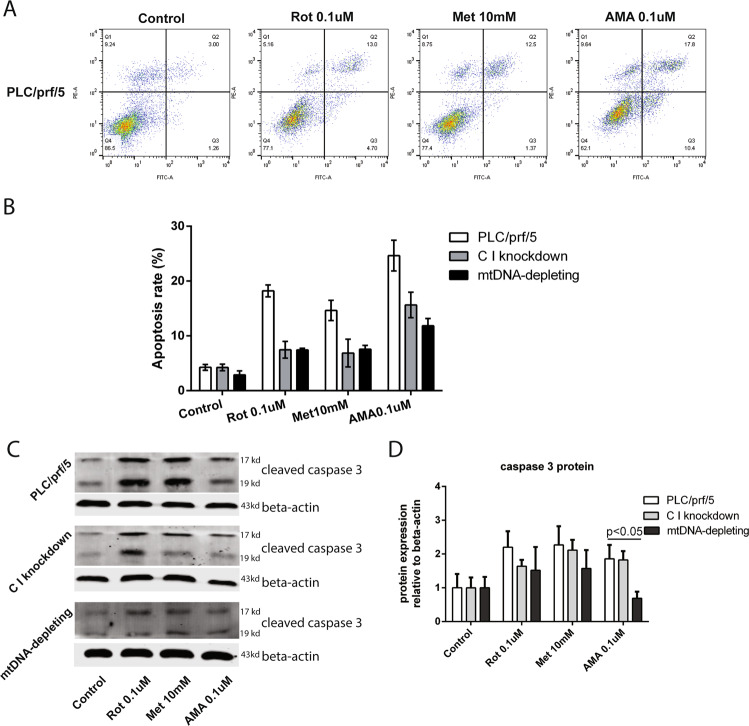
Inhibition of mETC complexes induced apoptosis. **A** Apoptotic cells were quantified by flow cytometry using Annexin V and propidium iodide co-staining in PLC/PRF/5 cells. The effects of treatment with ROT, Met or AMA for 48 h is shown (*N* = 3). **B** After treating cells with complex I and III inhibitors (ROT, Met, and AMA) for 48 h, the apoptotic rates of three HCC cell lines (PLC/PRF/5, C I knockdown, and mtDNA depleting) were quantified by flow cytometry. Results were presented as mean ± SD (*N* = 3). **C,** **D** Three conditions (PLC/PRF/5, C I knockdown, and mtDNA-depleted cells) are shown in presence or absence of ROT, Met, and AMA, following a 48 h incubation. The levels of cleaved caspase-3 protein were measured by the Western blotting analysis and quantified by the Image J software. Results were presented as mean ± SD, and group differences were tested by Mann–Whitney *U* test.

To further characterize the effects of targeting the mETC in liver cancer cells, we measured cellular apoptosis after treatment with complex I or complex III inhibitors. Programmed cell death can be studied using various approaches including measuring phosphatidylserine in the outer leaflet of the plasma membrane and the activation of caspases [18]. After treatments with mETC complex I inhibitors (ROT and Met) for 48 h, ~ 18–20% of PLC/PRF/5 displayed apoptosis measured by Annexin V staining, whereas cells that underwent depletion of mitochondrial DNA or knockdown of NDUFS1 showed the trend of less sensitive to inhibitor-induced apoptosis (Fig. 5A, B and supplementary Fig. S2). Similar results were observed in western blot assay(Fig. 5C, D). In contrast, NAC (N-acetylcysteine), the ROS inhibitor, cannot induce apoptosis in PLC/PRF/5 at the concentration of 10 mM (supplementary Fig. S3). These results, thus, suggested that mETC inhibitors may reduce liver cancer cell viability via increasing cell apoptosis.

### Inhibition of mETC complex I and III is associated with increased ROS production and decreased ATP generation

**Fig. 6:**
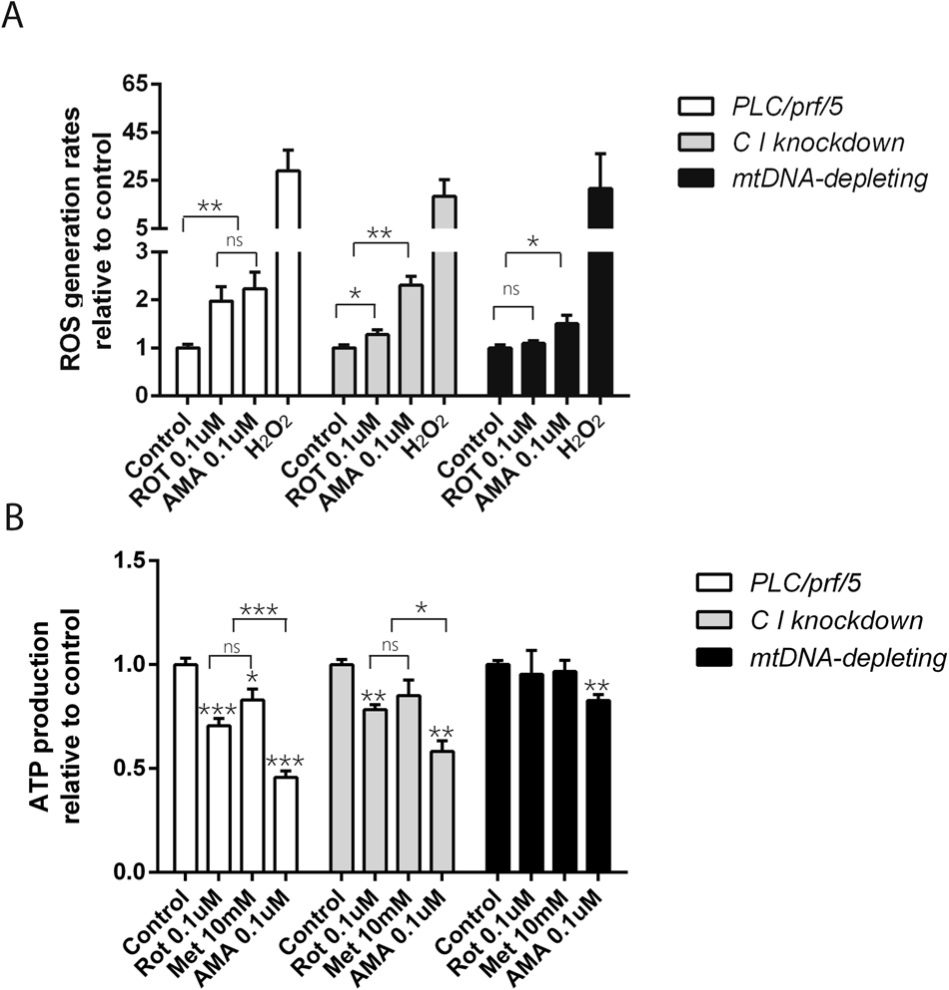
Inhibition of mETC affects cellular metabolism. **A** ROS production was measured after treating cells with ETC complex I (ROT) and III (AMA) inhibitors in three HCC cell lines (PLC/PRF/5 cells, C I knockdown, and mtDNA depletion) (*N* = 4). **B** After treating with ROT, Met, and AMA for 48 h, respectively, ATP production of PLC/PRF/5 cells, C I knockdown and mtDNA-depletion HCC cells were measured. (*N* = 9). Results were presented as mean ± SD, and group differences were tested by one-way ANOVA. *p < 0.05; ns, not significant.

The mETC is a major source of mitochondrial ROS generation as up to 2% of electrons leak from the mETC and subsequently react with oxygen in a one-electron reduction to produce ROS instead of water molecules [19]. It is well-conceivable that a defective mETC will result in excessive ROS generation, which can mediate downstream apoptosis. Therefore, we measured cellular ROS levels of HCC cells in presence or with absence of an mETC complex I inhibitor (ROT) and an mETC complex III inhibitor (AMA). In our experimental system, H_2_O_2_ induced marked ROS production and thus served as a positive control. Our results showed that both inhibition of complex I by ROT and complex III by AMA substantially increased cellular ROS levels. In the NDUFS1 knockdown cells or cells depleted for mitochondrial DNA, pharmacological inhibitors of the mETC did not show this effect (Fig. 6A). It thus appears that pharmacological inhibition of mETC complex I or mETC complex III is associated with increased ROS production.

To further characterize the functionality of the mETC in the physiology of liver cancer cells, we also measured cellular ATP levels. As expected, inhibition of the mETC diminished cellular ATP levels (Fig. 6B), decreased the pyruvate level involved in TCA, and disrupted NAD/NADH homeostasis, which indicated redox status (supplementary Fig. S4).

Collectively, our results revealed that the mETC complex I and mETC complex III played a pivotal role in liver cancer cell physiology.

## Discussion

Liver cancer is a major challenge to global health. Therapeutic options of liver cancer, particularly for advanced disease, remain unsatisfactory [20], and it is frustrating that liver cancer treatment is always accompanied with therapeutic drug resistance [21]. Increasing the understanding of liver cancer cell physiology may lead to novel therapeutic options, and the current study focused on the role of the mETC contributes to the increase. We showed that various strategies targeting mETC complex I and III limits the proliferation of liver cancer cells in cell lines, liver organoids, and xenografts in vivo.

Meanwhile, we found that inhibiting mETC disrupts the glycolytic pathway. As an essential redox carrier, NAD can accept hydrides produced along glycolysis and TCA; its reduced form, NADH, fuels mETC at the complex I level. NAD/NADH homeostasis is critical for cell survival [22, 23]. A decrease in the NAD/NADH ratio causes ROS to increase and dysregulate mETC [24–26]. Our study found the ratio of NAD/NADH content and pyruvate level decreased in normal organoids with mETC inhibitors treatment (supplementary Fig. S4). The results indicate that inhibiting mETC perturbed glycolysis and disrupted NAD/NADH homeostasis. However, the interaction between metabolic processes and cellular redox status in liver cancer remains unclear.

Mechanistically dysregulated mETC may be linked to cancer cell apoptosis, possibly provoked by increased ROS production following such inhibition. On the contrary, ROS inhibitors do not induce cell apoptosis in our experiments.

Although intuitively targeting mitochondrial metabolism appears implausible and potentially fraught with side effects, it is important to note that Metformin (which we also included in the present study) has already been clinically used as a complex I inhibitor [27]. Clinical experience with Metformin is especially extensive in patients with type II diabetes, and use of Metformin is associated with a reduced propensity to contract liver cancer, which is in apparent agreement with the effects observed by mETC inhibition in the current study [9, 28]. In general, the effects of Metformin in cancer are linked to AMPK/mTOR signaling [29]. To support this notion is the observation that inhibition of mETC complex I or mETC complex III is associated with reduced ATP levels, which is well established to provoke activation of AMPK and inhibition of mTOR [30]. Also in agreement with the idea is that activating mutations upstream of mTOR in Tuberous sclerosis complex (TSC) are regularly detected in liver cancer, suggesting that this pathway is indeed important for the development of liver cancer. However, in an unbiased kinome profiling study, our previous study found that the AMPK-provoked activation of TSC leads to the stimulation of p65PAK in addition to mTOR [31, 32].

Our study indicated inhibition of mETC complex I and mETC complex III resulted in increased ROS generation, which is reported as the underlying mechanism of limiting cancer cell growth [14, 33, 34]. However, the role of ROS production in cancer cell physiology is only partly understood [35, 36].

It is clear from the present study that targeting the mETC is a novel viable option for managing liver cancer, and our results provide a proof of concept that targeting mETC complex I or mETC complex III may be a potential novel avenue to combat liver cancer.

## Materials and Methods

### Reagents and antibodies

Rotenone (ROT), Metformin (Met), 2-Thenoyltrifluoroacetone (TTFA), Antimycin A (AMA), Myxothiazol (MYXO), puromycin, uridine, diphenyleneiodonium chloride, ethidium bromide (EtBr), and Potassium cyanide (KCN) were purchased from Sigma-Aldrich (St. Louis, MO). The rabbit polyclonal antibody against cytochrome b (CYTB) and mouse monoclonal antibody against NDUFS1 were purchased from Sigma-Aldrich (St. Louis, MO). The rabbit monoclonal antibody against cleaved caspase-3 was purchased from Cell Signaling Technology.

### Cell culture

Human cell lines HepG2, PLC/PRF/5, and Snu-449 were kindly provided from the Department of Viroscience, Erasmus Medical Center, and cultured in Dulbecco’s modified Eagle medium (DMEM) (Lonza Biowhittaker, Verviers, Belgium) supplemented with 10% (v/v) heat-inactivated fetal bovine serum (FBS, Thermo Fisher Scientific), 100 IU/ml penicillin and 100 IU/ml streptomycin. A mitochondrial DNA (mtDNA)-depleted cell model was established by co-culturing with EtBr (50 ng/ml), pyruvate (100 ug/ml), and uridine (50 ug/ml) for 5 days as described early [37]. For knockdown of mETC complex I, cells were transduced with an appropriate short hairpin RNA (shRNA), and transduced cells were selected by culturing with 3 ug/ml puromycin in DMEM according to previously described procedures [38]. The Plko.1-based shRNA lentiviral vectors sequence was: NDUFS1, 5'-CCGGGCAAGCAGATAGAAGGCCATACTCGAGTATGGCCTTCTATCTGCTTGCTTTTT-3'.

Organoids were derived and cultured as described early [39]. The study was approved by the medical ethical committee of Erasmus Medical Center. In addition, the study protocol conforms to the ethical guidelines of the 1975 Declaration of Helsinki.

### Proliferation assays of cells and organoids

Cell proliferation was measured by reduction of 3-(4,5-Dimethylthiazol-2-yl)-2, 5-diphenyltetrazolium bromide (MTT, MilliporeSigma) and Alamar Blue™ Cell Viability Reagent (Thermo Fisher, CA), essentially as described earlier [40]. In short, for the MTT assay, cells were seeded into a 96-well plate with or without mETC complex inhibitors and cultured for 48 h. Then, 15 ul of 10% MTT solution (5 mg/ml) per well was added to the wells followed by incubation at 37 °C in a 5% CO2 for 4 h. The medium was removed and 100 ul DMSO was added to each well and shaken for 30 min. The absorbance of each well was read on a microplate absorbance reader (Bio-Rad, Hercules, CA, USA) at a wavelength of 490 nm.

For the Alamar Blue assay, after treating the cells or organoids with inhibitors as appropriate for 48 h, the medium was removed and exchanged for fresh culture medium supplemented with 5% Alamar Blue and cultured at 37 °C with 5% CO2 for 4 h. Fluorescence (excitation 530 nm, emission 590 nm) of each well was measured on a FLUO STAR OPTIMA microplate reader (BMG Labtech, Durham, NC, USA).

### NAD/NADH assay

NAD and NADH content were measured by NAD/NADH Quantification Kit (Sigma-Aldrich, St. Louis, MO). PLC/PRF/5 or normal mouse organoids were treated with appropriate inhibitors for 48 h. Cells were seeded into a 6-well plate with or without mETC complex inhibitors and cultured for 48 h. Then cells were collected and extracted with 400 uL of NADH/NAD extraction buffer by freeze/thawing two cycles. Samples were centrifuged at 13,000 g for 10 min after 10 s vortex, then 200 ul supernatant was transferred to a fresh tube for NAD detection. The other was transferred into microcentrifuge tubes and heated to 60 °C for 30 min for NADH detection. A mixture of 50 ul samples and 100 ul reaction reagent was added into a 96-well plate to detect NAD and NADH content, respectively. The absorbance (450 nm) of each well was measured by the Infinite M Nano absorbance plate reader (Tecan, Switzerland).

For PLC/PRF/5, the cell concentration was 2 × 10^7^/ml. For organoids, the final results were normalized by total protein concentration.

### Pyruvate assay

Pyruvate level was measured by Pyruvate Assay Kit (Abcam, Cambridge, UK). Experiments were performed according to the manufacturer’s instructions. Cells were seeded into a 6-well plate with or without mETC complex inhibitors and cultured for 48 h. Then, cells were harvested and washed with cold PBS. Pyruvate Assay buffer was added to resuspend cells. The samples were centrifuged for 5 min at 4 °C at top speed. Subsequently, the supernatant of the samples were collected and transferred to clean tubes. A mixture of 50 uL reaction reagent was added to 50 uL supernatant. The mixtures were incubated at room temperature for 30 min and protected from light. The absorbance (570 nm) of each well was measured by the Infinite M Nano absorbance plate reader (Tecan, Switzerland).

For PLC/PRF/5, the cell concentration was 2 × 10^5^ /ml. For organoids, the final results were normalized by total protein concentration.

### Colony formation efficiency assay

After treatment of the cells with appropriate inhibitors, cells were cultured in a drug-free medium for ~ 14; days. The cells were fixed with methanol and stained with crystal violet for 1 hour, and then washed with water and dried before counting of colonies. A total number of colonies that contained more than 50 cells were counted, and the colony formation efficiency (CFE) was calculated. The results were normalized to the unexposed control (set to 100 % plating efficiency).

CFE% = (number of colonies in exposure cultures)/ (number of colonies in unexposed cultures) *100%

### Analysis of cell apoptosis

Cell apoptosis was measured by quantitatively determining cell surface phosphatidylserine in apoptotic cells using Annexin V-FITC/PI apoptosis detection kit (Becton Dickinson). Experiments were performed according to the manufacturer’s instructions. The treated cells were washed with cold PBS after co-culturing with the appropriate complex inhibitors for 48 h and suspended by 1X Binding Buffer. FITC Annexin V and PI (1:1) were added into 100 ul suspension (1 × 10^4^ cells) and incubated for 15 min at room temperature (25 °C) in the dark. The samples were tested by FACS within 1 hour [41]. Cell apoptosis rate was analyzed by FlowJo_V10 software. For each treatment, two independent wells were tested for PLC/PRF/5, C I knockdown, and mtDNA-depleting cell lines for three times. The mean and standard error were calculated for each condition.

### Xenograft mouse model in nude mice

The xenograft tumor model was established in female nude mice injected subcutaneously with knockdown cells and corresponding control cells into the lower left or right flank of the same mice (5 × 10^6^/200 uL cells per mouse; *n* = 5 mice per group), 1:1 mixed with matrigel. The xenograft mouse model was performed as described before [42]. All animal experiments were approved by the Committee on the Ethics of Animal Experiments of the Erasmus Medical Center.

### Reactive oxygen species measurements

Cellular reactive oxygen species (ROS) was measured using CM-H2DCFDA (General Oxidative Stress Indicator) kit (Thermo Fisher). Cell lines were seeded into 6-well plates and pretreated with diphenylene iodonium (DPI, 10 uM) for 1 h prior to appropriate complex inhibitor treatment for 3 h. The positive control was induced by adding 100 uM H_2_O_2_ for 15 min. Cells were washed with PBS and incubated with a non-serum medium containing 5 uM CM-H2DCFDA at 37 °C for 30 min. Then, the cells were washed with PBS for three times and suspended by 500 ul PBS. The samples were tested by a FACS, and ROS production was analyzed by FlowJo_V10 software.

### Adenosine triphosphate (ATP) production measurement

An ATP Bioluminescence Assay Kit HS II was used to measure the ATP content of cells according to the manufacturer’s instructions (Roche Life Science, Penzberg, Germany). Cells were seeded into a 96-well plate with or without mETC complex inhibitors and cultured for 48 h. Then, cells were harvested and suspended in dilution buffer at a concentration of 1 × 10^5^ /ml. The same volume of cell lysis reagent was added into to above cell suspension and incubated at 15 °C for 5 min and for an extra 2 min at 100 °C. Subsequently, the cell suspension was centrifuged at 10,000 g for 60 s and the supernatant was transferred to a fresh tube. Samples were kept on ice until measurement. A mixture of 50 ul luciferase reagent was added to 50 ul supernatant or standards provided by ATP Assay kit. Luminescence was detected after 1 s delay using a microplate reader (LumiStar Optima Luminescence Counter, BMG Labtech, Offenburg, Germany) (excitation = 535 nm; emission = 587 nm).

### Real-time quantitative polymerase chain reaction (PCR)

RNA was isolated with a Machery-NucleoSpin RNA kit (Bioke, Leiden, the Netherlands) and quantified with a Nanodrop ND-1000 (Wilmington, DE). The iScript cDNA synthesis kit (Takara Bio INC.) was used to acquire cDNA from total RNA. Quantitative real-time PCR analyses were performed by the StepOne Real-Time PCR System and the Step-Onev2.0 software (Applied Biosystem, Darmstadt, Germany). Primer sequences are provided in Supplementary Table 1. All the expression levels are depicted relative to the expression of GAPDH.

### Western blot assay

Western blotting was performed according to routine procedure [43]. In short, total protein (100 ug) was loaded in each lane, subjected to sodium dodecyl sulfate-polyacrylamide (SDS-PAGE) gel (12%) electrophoresis and then transferred onto polyvinylidene difluoride (PVDF) membranes (Invitrogen). Subsequently, the membranes were blocked for 1 hour at room temperature followed by incubation with antibodies overnight at 4 °C. Membranes were washed 3 times before incubating with secondary antibodies for 2 h. Protein bands were detected with Odyssey 3.0 Infrared Imaging System (LI-COR Biosciences) after washing 3 times.

### Statistical Analysis

All data are presented as mean ± SD. Prism software (GraphPad Software) was used for all statistical analyses. *N* = repeated times of experiments. Statistical analysis was performed one-way ANOVA if multi-group of samples were analyzed; otherwise Mann–Whitney test was performed. For all experiments, a *p*-value < 0.05 was considered as significant.

## Supplementary information

Supplementary materials

Figure s1

Figure s2

Figure s3

Figure s4

## Data Availability

The data are available upon reasonable request.
