# Supplementary material for: Targeting the complex I and III of mitochondrial electron transport chain as a potentially viable option in liver cancer management
Source: Cell Death Discov. 2021 Oct 14;7:293. doi: 10.1038/s41420-021-00675-x (PMC8516882; doi:10.1038/s41420-021-00675-x)
Supplement: Supplementary file 1 — Supplementary materials [file 41420_2021_675_MOESM1_ESM.docx]

Supplementary data

**Targeting the complex I and III of mitochondrial electron transport chain as a potential viable option in liver cancer management**

Qin Yang, Ling Wang, Jiaye Liu, Wanlu Cao, Qiuwei Pan and Meng Li

**Table of contents**

Supplementary Figure 1

Supplementary Figure 2

Supplementary Figure 3

Supplementary Figure 4

Supplementary Table 1

**Figure s1. The half maximal inventory concentration curve of complex I and III inhibitors.**

Cell viability was quantified by MTT and AlamarBlue.

**Figure s2. The apoptosis rate in complex I-knockdown and mtDNA-depleting cells of PLC/PRF/5.** Apoptotic cells were quantified by flow cytometry with Annexin V and propidium iodide co-staining in complex I-knockdown and mtDNA-depleting cells of PLC/PRF/5 after 48h mETC inhibitors treatments.

**Figure s3. The apoptosis rate in different cell lines.**

A-C. Apoptotic cells were quantified by flow cytometry, staining with Annexin V and propidium iodide, in PLC/PRF/5 cells, complex I-knockdown and mtDNA-depleting cells of PLC/PRF/5 with/without NAC treatment. D. After treating cells with 10mM NAC for 24 hours or mETC inhibitors for 48 hours, respectively, the apoptotic rates of three HCC cell lines (PLC/PRF/5, C I knockdown and mtDNA-depleting) were quantified by flow cytometry.

**Figure s4. The alternations of glycolytic pathway and redoxd status after mETC inhibitors treatment.**

A-B.NADH level of mouse normal liver organoids and PLC cell lines, respectively. C-D. The ratio of NAD/NADH in mouse normal liver organoids and PLC cell lines, respectively. E-F. Pyruvate level of mouse normal liver organoids and PLC cell lines, respectively.

**Table s1** Primers sequences used for qRT-PCR

| Gene | Forward sequence (5’-3’) | Reverse sequence (5’-3’) |
| --- | --- | --- |
| NDUFS1 | TGTGTGAGACGGTGCTGATGGA | CGATGGCTTTCACGATGTCCGT |
| COX I | CCTGACTGGCATTGTATTAG | GATAGGATGTTTCATGTGGTG |
| COX II | CATCCCTACGCATCCTTTAC | GGTTTGCTCCACAGATTTCAG |
| COX IV | CAGAAGGCACTGAAGGAGAAG | TCATGTCCAGCATCCTCTTG |
| CYTB | CCCTAACAAACTAGGAGGCG | TCTGCGGCTAGGAGTCAATA |
| GAPDH | GGAAATCCCATCACCATCT | GGACTCCACGACGTACTCA |
