# Supplementary figures and images for: Targeting the complex I and III of mitochondrial electron transport chain as a potentially viable option in liver cancer management
Source: Cell Death Discov. 2021 Oct 14;7:293. doi: 10.1038/s41420-021-00675-x (PMC8516882; doi:10.1038/s41420-021-00675-x)

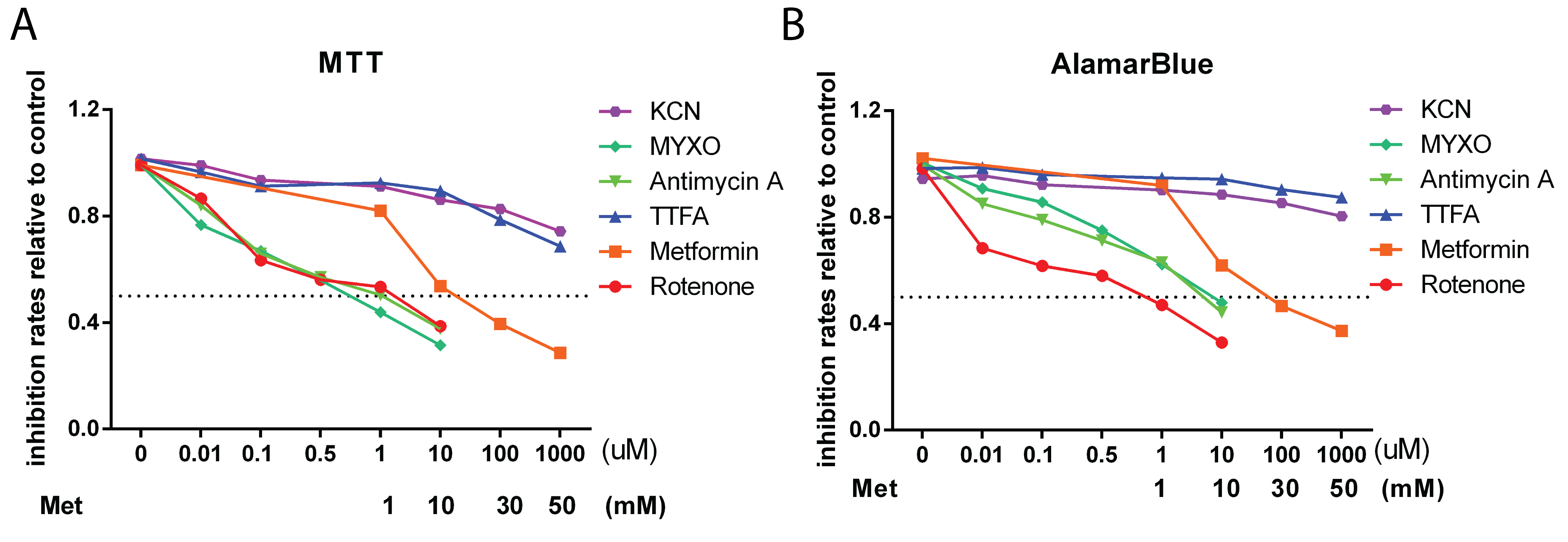

Supplement: Supplementary file 2 — Figure s1 [file 41420_2021_675_MOESM2_ESM.tif]

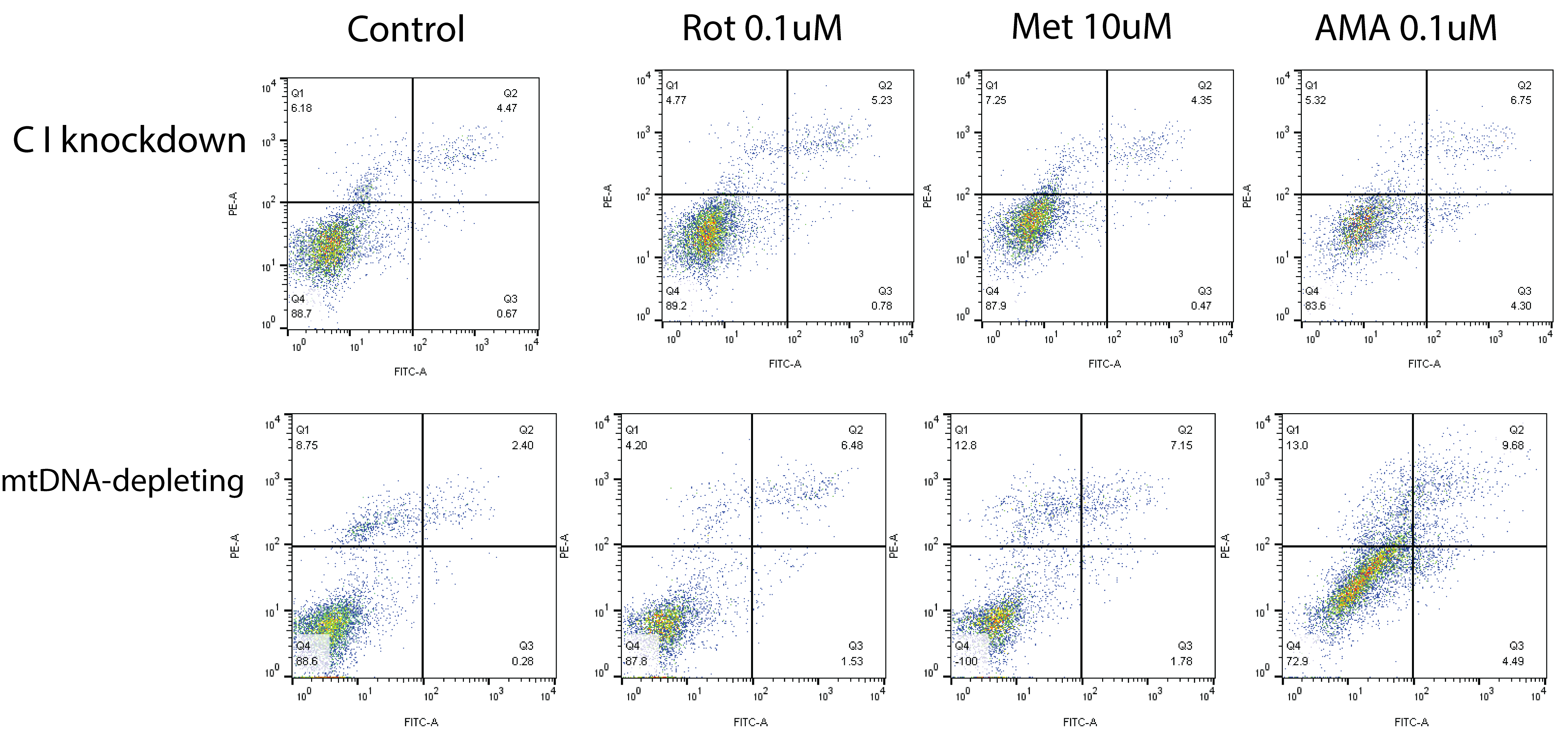

Supplement: Supplementary file 3 — Figure s2 [file 41420_2021_675_MOESM3_ESM.tif]

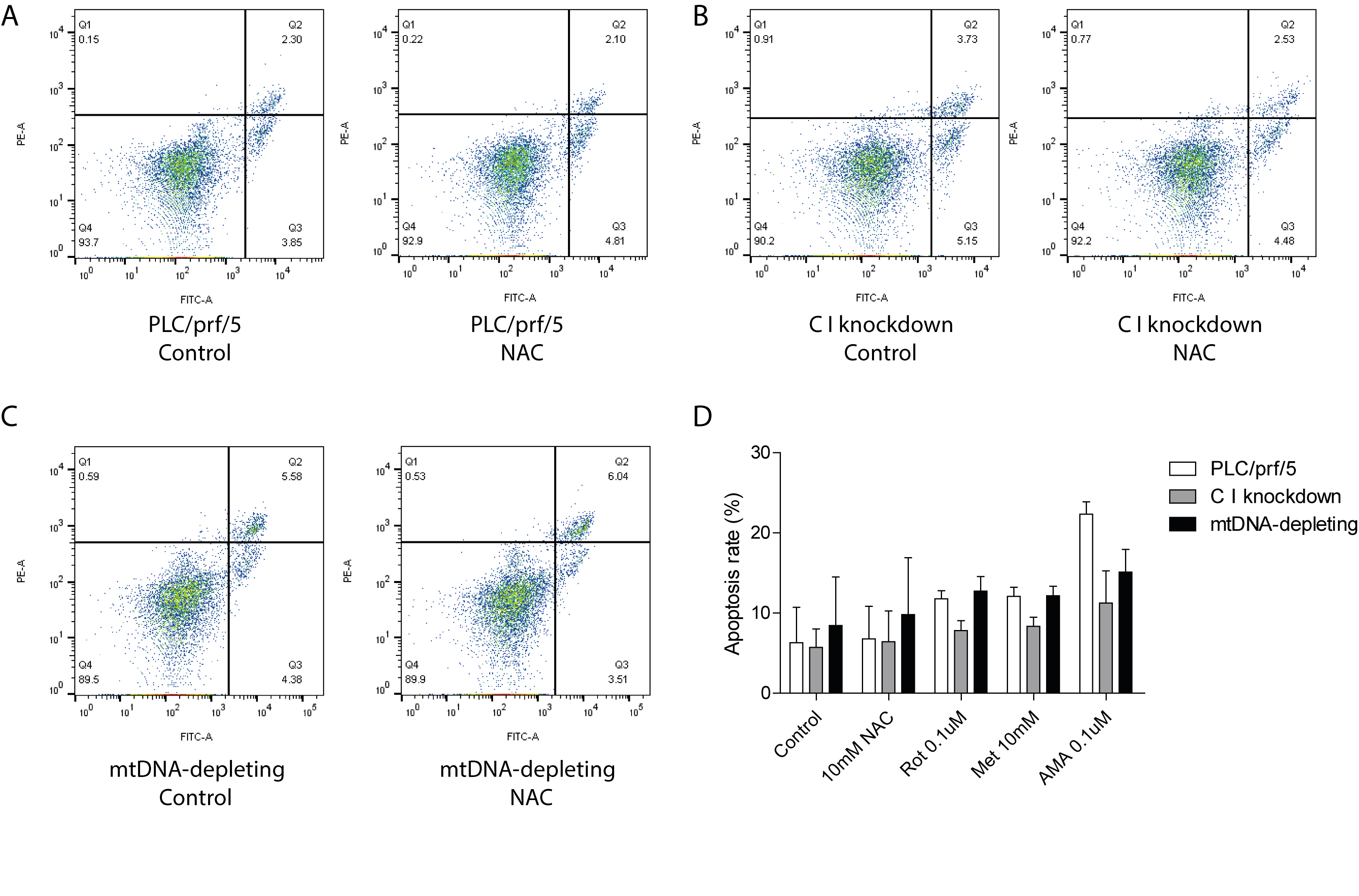

Supplement: Supplementary file 4 — Figure s3 [file 41420_2021_675_MOESM4_ESM.tif]

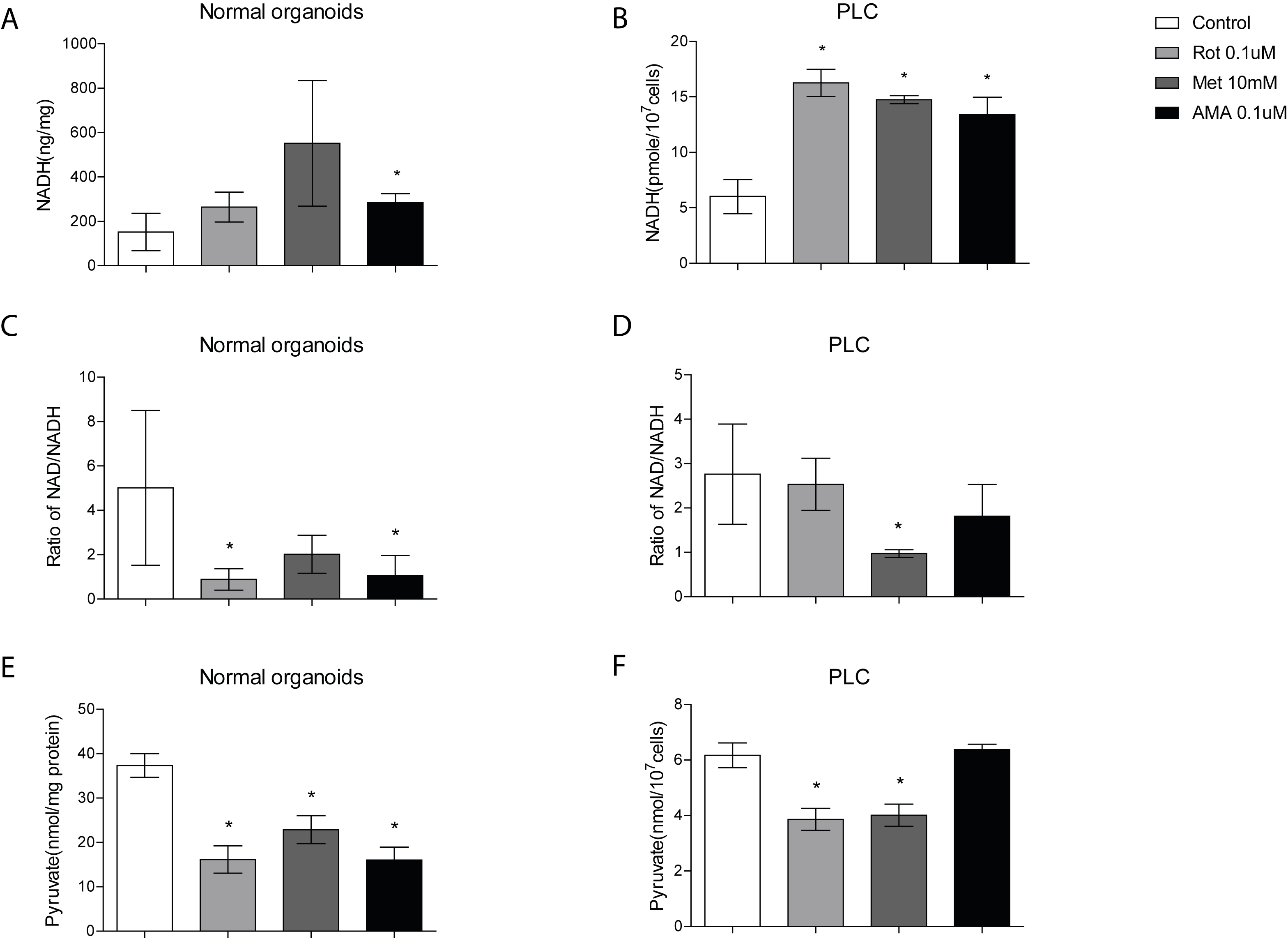

Supplement: Supplementary file 5 — Figure s4 [file 41420_2021_675_MOESM5_ESM.tif]
